# Differential diagnosis of posterior fossa brain tumors: Multiple discriminant analysis of Tl-SPECT and FDG-PET

**DOI:** 10.1097/MD.0000000000007767

**Published:** 2017-08-18

**Authors:** Moritaka Yamauchi, Tomohisa Okada, Tsutomu Okada, Akira Yamamoto, Yasutaka Fushimi, Yoshiki Arakawa, Susumu Miyamoto, Kaori Togashi

**Affiliations:** a Department of Diagnostic Imaging and Nuclear Medicine; b Department of Neurosurgery, Kyoto University Graduate School of Medicine, Kyoto, Japan.

**Keywords:** brain tumor, discriminant analysis, FDG, PET, SPECT, thallium-201

## Abstract

This study investigated the combined capability of thallium-201 (Tl)-SPECT and fluorine-18-fluoro-deoxy-glucose (FDG)-PET for differential diagnosis of posterior fossa brain tumors using multiple discriminant analysis.

This retrospective study was conducted under approval of the institutional review board. In the hospital information system, 27 patients with posterior fossa intra-axial tumor between January 2009 and June 2015 were enrolled and grouped as the following 7 entities: low grade glioma (LGG) 6, anaplastic astrocytoma (AA) 2, glioblastoma (GBM) 3, medulloblastoma (MB) 3, hemangioblastoma (HB) 6, metastatic tumor (Mets) 3, and malignant lymphoma (ML) 4. Tl and FDG uptakes were measured at the tumors and control areas, and several indexes were derived. Using indexes selected by the stepwise method, discriminant analysis was conducted with leave-one-out cross-validation.

The predicted accuracy for tumor classification was 70.4% at initial analysis and 55.6% at cross-validation to differentiate 7 tumor entities. HB, LGG, and ML were well-discriminated, but AA was located next to LGG. GBM, MB, and Mets largely overlapped and could not be well distinguished even applying multiple discriminant analysis. Correct classification in the original and cross-validation analyses was 44.4% and 33.3% for Tl-SPECT and 55.6% and 48.1% for FDG-PET.

## 1 Introduction

In most of the brain tumors in the posterior fossa, contrast enhancement is frequently observed, and differential diagnosis using computed tomography (CT) or magnetic resonance imaging (MRI) is not always feasible. For differential diagnosis, imaging with radioisotopes, which are considered to reflect tumor functions and characteristics, is also a method of choice. Thallium-201 (Tl) single photon emission computed tomography (SPECT) imaging has long been used for brain tumors, and more recently, fluorine-18-fluoro-deoxy-glucose (FDG) positron emission tomography (PET) is also widely used in clinical practice. Both SPECT and PET can characterize and measure biologic processes at the cellular and molecular levels and provide different additional biochemical or molecular information about brain tumors.^[1]^ In most of the cases, these 2 scan methods have been investigated separately, and several studies investigated combined diagnostic capability using discriminant analysis.^[2,3]^

Discriminant analysis is a statistical approach used to predict a categorical dependent variable by 1 or more continuous or binary independent variables. This analysis works by creating 1 or more linear combinations of predictor variables that provide the best discrimination between the groups: these are called discriminant functions. In the field of image analysis, multiple discriminant analysis (MDA) has become much used in clinical practice. This method has successfully applied to grading gliomas^[4]^ and survival prediction of high-grade glioma after recurrence.^[5]^ It was also used for differential diagnosis of dementia^[3]^ and Parkinsonism.^[6]^

There has been many PET/SPECT imaging studies on the brain tumors,^[7–18]^ either only for Tl-SPECT ^[7,9,10,15–18]^ or FDG-PET.^[8,11,12]^ Even in studies using both of Tl-SPECT and FDG-PET, they investigate either within astrocytic tumors of the whole brain^[14]^ or extra-axial meningiomas^[13]^ without combined analysis. No previous report exists that investigated specifically for the tumors in the posterior fossa. In this study, we evaluated combined analysis of 2 major radioisotope imaging of Tl-SPECT and FDG-PET to differentiate posterior fossa tumors by applying MDA.

## 2 Materials and methods

### 2.1 Patients

This retrospective study was conducted under approval by the institutional review board, and informed consent was waived. We searched institutional patient database between January 2009 and June 2015 for intra-axial tumors in the posterior fossa with both Tl-SPECT and FDG-PET imaging. In total, 27 patients were found and classified into the following 7 groups: low grade glioma (or LGG, hereafter) 6 patients, anaplastic astrocytoma (AA) 2, glioblastoma (GBM) 3, medulloblastoma (MB) 3, hemangioblastoma (HB) 6, metastatic tumors (Mets) 3, and malignant lymphoma (ML) 4. Histopathological diagnosis was obtained by surgical resection or biopsy for all cases except 4 LGG cases that were diagnosed by imaging findings and stable clinical courses (Table 1).

**Table 1:**
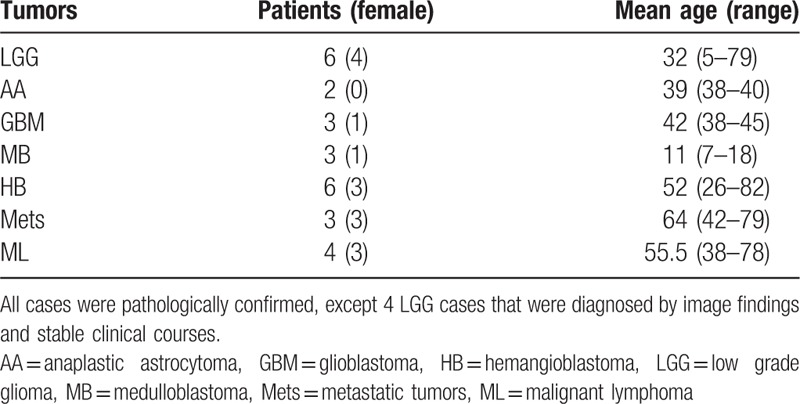
Tumor classification.

### 2.2 Image acquisition

A Tl-SPECT scan was conducted using a 2-head rotating gamma camera (Infinia; GE Medical Systems, Milwaukee, WI) with extended low-energy general-purpose collimators. After intravenous administration of 74 MBq of Tl, the scan was conducted at 15 minutes after the injection. Data were acquired through a 360° rotation at angle intervals of 6°, each for 20 seconds. Total imaging time was 20 minutes. Transverse reconstruction was conducted using ordered subset expectation maximization (subsets: 10 and iterations: 2), and resolutions were 4.42 mm × 4.42 mm × 4.42 mm (33–47 slices) with in-plane matrix of 64 × 64 and field of view of 282.9 × 282.9 mm.

FDG-PET images were acquired with a PET/CT scanner (Discovery ST Elite; GE Healthcare, Waukesha, WI). Patients fasted for at least 4 hours prior to the scan. After intravenous administration of 4 MBq/kg of FDG, patients rested in a waiting room for 30 minutes. An emission scan of the brain was conducted for 15 minutes. Resolutions were 2.0 mm × 2.0 mm × 4.25 mm (47 slices) with the in-plane matrix of 128 × 128 and field of view of 256 × 256 mm.

As an anatomical reference, MR scans were conducted using 3T MR units (Magnetom Trio or Magnetom Skyra; Siemens, Erlangen, Germany) with a 32-channel head coil. Preoperative scans included 3-dimensional T1-weighted imaging of isotropic 0.9 mm resolution covering the whole brain before and after administration of a Gadolinium contrast agent (0.1 mmol/kg). Parameters of MPRAGE were TR/TE, 1900/2.58 ms; inversion time, 900 ms; flip angle, 9°; FOV, 230 × 230 mm; matrix size, 256 × 256; slab thickness, 208 mm partitioned into 0.9 mm; parallel imaging factor, 2 in phase encoding direction; and imaging time 4 min 26 s.

### 2.3 Image analysis

Uptake of Tl and FDG in a tumor was evaluated using semi-quantitative analysis by placing a region of interest (ROI) within the tumor. It was placed manually by an evaluator (MY with 8 years of experience in diagnostic radiology) on the axial slice with the largest uptake, avoiding necrotic or cystic areas in the tumors with reference to the MR images. If there was only unclear uptake in the tumor, an ROI was placed with reference to the corresponding MRI image. In the case of multiple tumors, the largest or histologically proven lesion was selected for the analysis. The mean and maximum uptake values of Tl-SPECT (TU_mean_ and TU_max_, respectively) were normalized as ratios (TUR_mean_ and TUR_max_) by those of reference ROIs placed at the frontoparietal normal-appearing white matter of the ipsilateral side (TU_w_). The mean and maximum uptake values of FDG-PET (SUV_max_ and SUV_mean_) were normalized as ratios by those of reference ROIs (SUV_w_ and SUV_g_) placed at the frontoparietal normal-appearing white matter (SUVR_max/w_ and SUV_mean/w_) and gray matter (SUVR_max/g_ and SUR_mean/g_) of the ipsilateral side. When the tumor was found in the median line, they were placed at normal appearing areas on either side of MR images as in previous studies.^[11,19]^ The measurement of TU_mean_, TU_max_, TU_w_, SUV_max_, SUV_mean_, SUV_w_, and SUV_g_ were measured twice with interval of 1 month, so that the evaluator should not remember the former ROI placement and measurement should be independent of each other.

### 2.4 Statistical analysis

The analysis was conducted with SPSS version 23.0 (IBM Software, NY). For the measured values, that is, TU_mean_, TU_max_, TU_w_, SUV_max_, SUV_mean_, SUV_w_, and SUV_g_, intra-class correlation coefficients (ICC) were calculated between 1st and 2nd measurements to illustrate reliability of ROI measurements. The values were averaged and used for calculating tumor-to-normal ratios.

As the initial analysis, TUR_mean_, TUR_max_, SUV_max_, SUVR_max/w_, SUVR_mean/w_, SUVR_max/g_, and SUVR_mean/g_ were used as multiple variables for MDA to discriminate the 7 tumor groups. Stepwise discriminant analysis was carried out to select variables for optimal discrimination. After generating discriminant functions, cross validation was conducted for all cases to evaluate the differential efficacy. For each of the selected variables, discrimination accuracy was also evaluated.

## 3 Results

As for reproducibility of the ROI analysis, ICCs were 0.854 (95% CI 0.709–0.931), 0.783 (0.583–0.895), 0.873 (0.743–0.940), 0.979 (0.956–0.9991), 0.940 (0.874–0.972), 0.871 (0.740–0.939), and 0.970 (0.936–0.980) for TU_mean_, TU_max_, TU_w_, SUV_max_, SUV_mean_, SUV_w_, and SUV_g_, respectively, showing high reproducibility. The measured mean and standard deviation values are summarized in Table 2.

**Table 2:**
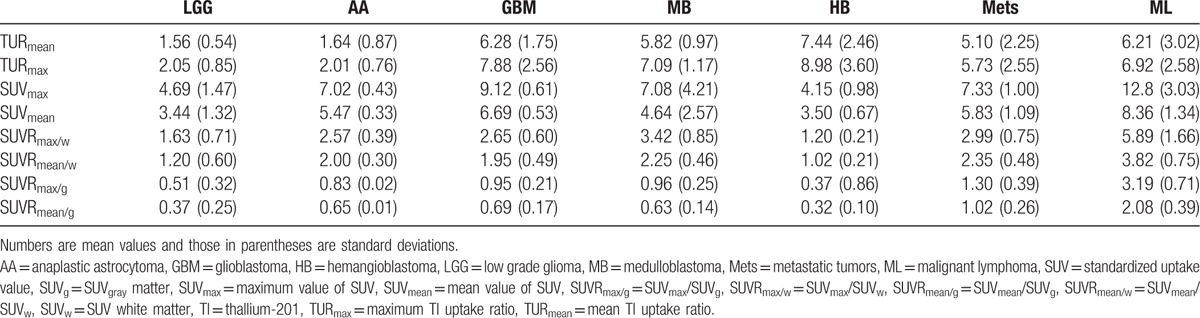
The average and standard deviation values for the tumor groups.

By stepwise discriminant analysis, TUR_mean_ and SUVR_max/g_ were selected, and 2 discrimination functions were constructed, describing 83.9% (discriminant function1, or DF1) and 16.1% (DF2) of the total variance, respectively. DF1 was constructed with linear combination of TUR_mean_ × (–0.279) and SUVR_max/g_ × 1.050. That of DF2 was linear combination of TUR_mean_ × 1.013 and SUVR_max/g_ × (–0.046).

**Figure 1:**
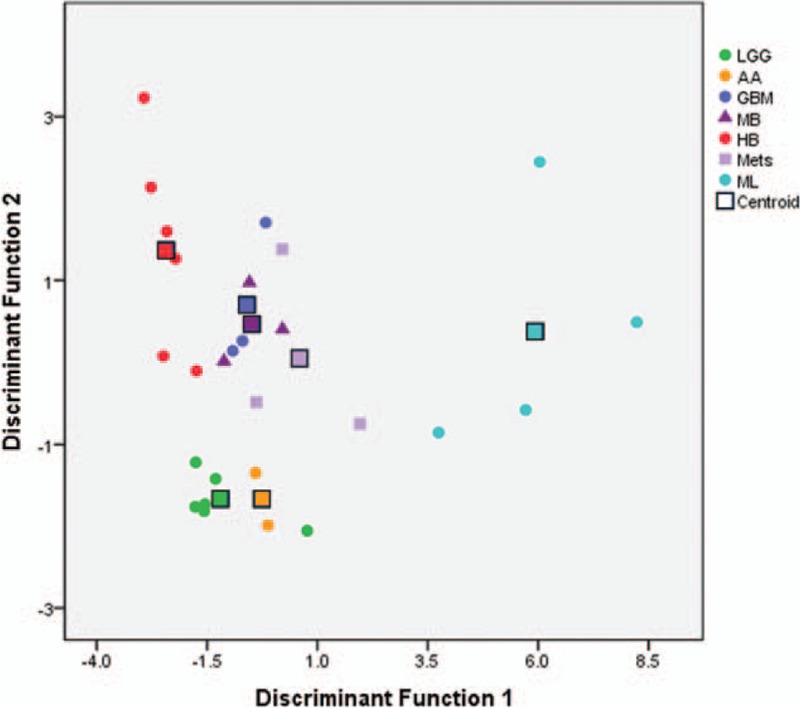
Results of multiple discriminant analysis. The 7 different tumors are presented in different colors. The round marks represent each patient, and the rectangles indicate centroids. LGG, HB, and ML are well separated, although LGG and AA are closely located at low values in both axes. GBM, MB, and Mets are found inseparable. For standard deviations, refer to Table 2. Abbreviations: AA = anaplastic astrocytoma, GBM = glioblastoma, HB = hemangioblastoma, LGG = low-grade glioma, MB = medulloblastoma, Mets = metastatic tumors, ML = malignant lymphoma.

By using these 2 variables, 70.4% of the original grouped cases and 55.6% of cross-validated grouped cases were correctly classified into 7 different tumor entities (see Table 3). Five of 6 cases of both HB and LGG were correctly discriminated in original as well as cross-validation analysis using combination of the 2 variables. All 4 ML cases were correctly classified in original grouped cases, but cross-validation found 1 misclassification. All AA cases were correctly classified as AA, but 1 LGG and 1 metastatic tumor were classified as AA, and predictive accuracy of AA is diminished. As in Fig. 1, GBM, MB, and Mets cases were overlapped and could not be well distinguished even applying MDA.

**Table 3:**
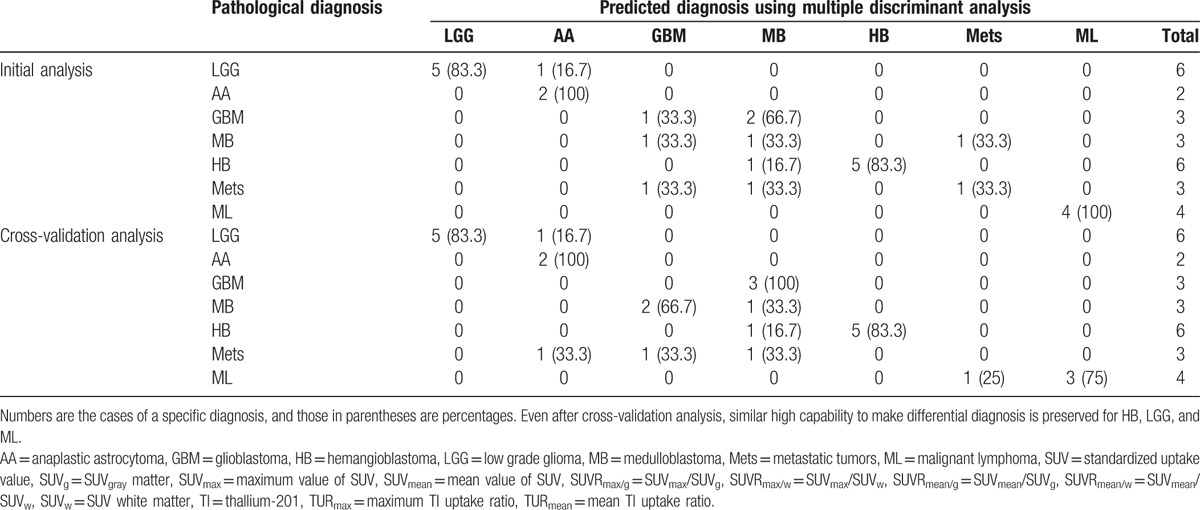
Results of discriminant analysis of the posterior fossa tumors in the initial analysis as well as its cross-validation results.

For each of the selected variables, correct classification of the original grouped cases and cross-validated grouped cases were 44.4% and 33.3%, respectively, for TUR_mean_, and 55.6% and 48.1% for SUV_max/g_.

## 4 Discussion

In this study, we evaluated the capability of a discriminant analysis for combined Tl-SPECT and FDG-PET for diagnosis of posterior fossa brain tumors. Using this method, the total diagnostic accuracy was not so high, but LGG, HB, and ML cases were almost correctly identified. Other tumors of AA, GBM, MB, and Mets could not be clearly discriminated.

From the viewpoint of therapeutic options, confirmation of LGG ensures follow-up observation. AA may require more intense therapeutic intervention than LGG, but AA has better prognosis than GBM. In ML, minimally invasive biopsy is required to confirm pathology and chemotherapy will be conducted for better prognosis.^[20]^ Hemangioblastoma can be controlled by stereotactic radiosurgery in 79% to 92% of tumors.^[21]^ Correct diagnosis helps to adopt appropriate therapy.

In other tumors that could not be classified correctly, MB is found mostly in children, which is highly different from others.^[22]^ In about 80% of patients with metastatic tumor, primary lesions were detected using whole-body FDG-PET.^[23]^ If a whole-body FDG-PET examination is available, differentiation would be much improved. After these procedures, what remains is GBM that is mostly found in relatively aged subjects. Additional clinical information helps to differentiate these tumors.

Stepwise discriminant analysis extracts best discriminant variables separating tumor categories, which enters or removes variables by analyzing their effects on the discrimination, and found 2 variables of TUR_mean_ and SUV_max/g_. When classification was compared between the 2 variables, SUV_max/g_ had higher capability than TUR_mean_. However, this capability was much augmented when MDA was applied. Although cross-validation found only 1 discrimination failure in ML, MDA retained relatively high diagnostic capability of LGG, HB, and ML. In MDA, DF1 explained 83.9% of the total variance, whereas DF2 explained 16.1%. DF1 was more weighted on SUVR_max/g_ than TUR_mean_ compared with DF2. What are the differences between them?

Tl is a potassium analog with high affinity to the sodium- and potassium-activated adenosine triphosphatase (Na+-K+ ATPase) pump. Its uptake in tumor cells can be explained by its mechanism of action, which is related to disruption of the blood–brain barrier, regional blood flow, and tumor cell uptake via Na-K-ATPase pump activity.^[24,25]^ Tl-SPECT has been used widely for the imaging of various brain tumors and to assess tumor viability. Kaplan et al^[26]^ reported that Tl imaging offered the most accurate correlation with viable tumors histologically in malignant glioma. However, Tl uptakes occur not only in biologically malignant tumors but also in benign tumors, such as meningiomas, pituitary adenomas, and HB.^[27–30]^ Therefore, it is difficult to estimate the grade of malignancy only from Tl uptakes.

On the other hand, FDG-PET uptake derives from glucose metabolism of tumor cells and their density. FDG is a glucose analogue that is transported from the blood into cells by glucose transporters (predominantly Glucose Transporter 1, or GLUT1). Once in the cell, FDG is phosphorylated by hexokinase (mainly HK2) to form FDG-6-phosphate. Further metabolism of FDG-6-phosphate cannot be conducted, and FDG-6-phosphate is essentially trapped in the cell. Significantly elevated GLUT1 and GLUT3 expression levels are considered to be responsible for the accumulation of FDG in malignant tumor.^[24,31,32]^ Furthermore, hexokinases are involved in glucose metabolism and the expression of these proteins may be correlated with FDG uptake. These mechanisms of uptake are different between Tl and FDG, and contributed to differentiation of the posterior fossa tumors.

HB is a highly vascular benign tumor with characteristic findings of a cerebral cystic region and a peripherally enhanced nodule. Its FDG uptake is relatively low,^[33]^ whereas Tl uptake is increased at early phase.^[15,34]^ This low and high uptake enables accurate discrimination of HB. ML is characterized by high cellular density and accelerated glycolytic metabolism and results in higher FDG uptake than GBM or Mets,^[11,12,35]^ although Tl uptakes are comparable among them.^[14]^ In LGG, uptakes of both Tl and FDG were low, and combined analysis discriminated LGG from HB. On the other hand, GBM, MB, and Mets had similar uptakes of both Tl and FDG, and discrimination was not much feasible.

There are several limitations in this study. First, we have collected a small sample of the posterior fossa brain tumors and results need further validation by prospective studies with larger sample size. Second, we could not include the other brain tumor (pilocytic astrocytoma, ependymoma, etc.) nor nontumorous lesions (inflammation, demyelination, and subacute infarction). Third, we used ROI-based analysis in this study. ROIs were operator-dependent. Fourth, in our study, only early scan of Tl-SPECT was conducted. In Tl imaging, a delayed scan at 3–4 hours after infusion is reported useful for evaluating tumor malignancy,^[17]^ but it imposes very long waiting time on patients and the performance of Tl dynamic SPECT at 15 minutes after infusion was reported to have high capability to distinguish malignant brain tumors from benign ones.^[15,34]^

In conclusion, discriminant analysis for Tl and FDG uptake tumor-to-normal uptake ratio has limited capability of differential diagnosis of the posterior fossa tumors, but can help to diagnose hemangioblastoma, lymphoma, and low-grade glioma.
